# Hemoporfin-Mediated Photodynamic Therapy for Port-Wine Stains: Multivariate Analysis of Clinical Efficacy and Optical Coherence Tomography Appearance

**DOI:** 10.3389/fmed.2022.800836

**Published:** 2022-02-24

**Authors:** Yanyan Lin, Wei Gong, Jie Kang, Yuhong Fang, Jingjing Liu, Lihang Lin, Xuemin Xiao

**Affiliations:** ^1^Department of Dermatology, The Union Hospital, Fujian Medical University, Fuzhou, China; ^2^Key Laboratory of OptoElectronic Science and Technology for Medicine of Ministry of Education, College of Photonic and Electronic Engineering, Fujian Normal University, Fuzhou, China; ^3^Department of Dermatology, Dermatology Hospital of Fuzhou, Fuzhou, China

**Keywords:** hematoporphyrin monomethyl ether (HMME), photodynamic therapy (PDT), port-wine stains (PWS), efficacy analysis, optical coherence tomography (OCT)

## Abstract

**Background:**

Hemoporfin-mediated photodynamic therapy (HMME-PDT) is reported to be effective and safe for port-wine stains (PWS). However, its efficacy is influenced by several factors and there is no appropriate method to evaluate efficacy so far. Therefore, this study explored the clinical efficacy of HMME-PDT for PWS on the face and neck and the feasibility of evaluating treatment potency with optical coherence tomography (OCT).

**Methods:**

A total of 211 PWS patients subjected to HMME-PDT were recruited for study and correlations of therapeutic effect with treatment sessions, age, gender, lesion distribution and treatment history analyzed. OCT was utilized for quantitative analysis of PWS lesions of 36 selected patients before and after HMME-PDT.

**Results:**

The efficacy of two consecutive treatments was significantly higher than that of single treatment (*P* < 0.05). In multivariate analysis, after the first treatment, age, lesion distribution and treatment history were correlative factors affecting treatment efficacy (*P* < 0.05). The improvement effect on central facial lesions was lower than that on lateral facial lesions (*P* < 0.05). The efficacy of therapy on the group with no history of pulsed dye laser (PDL) treatment was greater than that on effective and ineffective treatment groups (*P* < 0.05). After the second session, age remained the only factor correlated with efficacy (*P* < 0.05). Dilated vessel diameter and depth before and after treatment were significantly different (*P* < 0.05). With increasing treatment times, age was the most significant factor influencing treatment efficacy.

**Conclusions:**

Our collective findings indicate that HMME-PDT therapy is effective and safe for PWS and support the utility of OCT in objective assessment of the efficacy of HMME-PDT.

## Introduction

Port-wine stains (PWS) are a type of congenital and progressive telangiectasia or posttelangiectasia capillary malformation in 0.3–0.5% infants born worldwide (1). The stains initially appear as pinkish patches that may range from red to purple with varying degrees of hypertrophy or nodule formation with age, causing spontaneous bleeding and hemorrhage upon injury.

The pathogenesis of PWS is yet to be established and potentially associated with somatic genetic mutations (GNAQ, PI3K), MAPK and PI3K aberrant activations, molecular phenotypes of PWS endothelial cells, overexpression of vascular endothelial growth factor (VEGF) and its receptor VEGFR, formation of an immature venule-like vascular system and gradual dilation causing vascular malformation (2). PWS is characterized by ectatic capillaries 10–150 μm in diameter and located predominantly in the upper dermis at a depth of 300–600 μm (3). Approximately 70–80% of lesions occur on the face and neck (4), often leading to sociopsychological problems due to disfigurement and cosmetic concerns.

The current mainstream treatments include pulsed dye laser (PDL) and vascular targeted photodynamic therapy (PDT). PDL achieves therapeutic efficacy by destroying capillary malformation through selective photothermolysis and remains the gold standard of treatment at present. While a number of previous studies support beneficial effects of PDL on superficial PWS, the basal healing rate is only 6% and recurrence or redarkening after treatment are frequently reported (5).

Photodynamic therapy has undergone rapid development and been validated as an effective and safe alternative to PDL in accumulating clinical studies (6, 7). In 2016, a novel porphyrin-related photosensitizer, hematoporphyrin monomethyl ether (HMME), with several unique characteristics, such as short phase of light avoidance, short half-life, high safety, high selection of action sites, and a strong photodynamic effect, was successfully applied in as part of PDT in the clinic (8).

Earlier research on the effects of HMME-PDT on human umbilical vein endothelial cells disclosed apoptosis of ectatic vascular endothelial cells and inhibition of autophagy (9). After intravenous injection, HMME immediately reaches peak concentrations in blood and is absorbed rapidly by vascular endothelial cells but rarely by epidermal cells. The photosensitizer displays significant concentration differences between vascular endothelial and epidermal cells. Provision of light irradiation at 532 nm excites the photosensitizer to generate a photochemical reaction and produce singlet oxygen and other toxic substances that cause swelling, degeneration and necrosis of vascular endothelial cells and achieve therapeutic effects with no damage to the normal epidermal layer (10).

Optical coherence tomography (OCT) is a powerful non-invasive tool for imaging of skin structures. Based on the principle of light reflection delay and interference imaging, OCT can be efficiently used to image the internal structures of tissues. Scanning of optical scattering media, such as biological tissues, can provide morphological images of living tissues with micron resolution (11). Based on the long wavelength selected, light passes through a certain depth of scanning medium (with a maximum detection depth of up to 2 mm) (11), which can be used to identify most pathological changes in skin. Additionally, the imaging speed of OCT is <30 s, thus achieving high resolution and high-speed imaging. A number of previous studies have successfully employed OCT for examination of skin vascular structures (12, 13).

In 1991, Huang et al. (14) first proposed the concept of OCT and successfully used the technique to image microstructures in the retina and coronary artery wall of the human eye. The feasibility of OCT in the field of dermatology has additionally been confirmed in several inflammatory skin diseases, in particular, psoriasis and contact dermatitis, autoimmune skin disorders, such as scleroderma and autoimmune blistering disease (15), and vascular characteristics of sensitive skin (13).

PWS is characterized by obvious vascular hyperplasia, vascular dilation and shallow lesion distribution. Thus, OCT presents a suitable imaging modality for PWS. Hemoporfin-mediated photodynamic therapy (HMME-PDT) is reported to be effective and safe for PWS. However, its efficacy is influenced by several factors. In the current study, multivariate analysis was performed on a large scale to explore the impact of independent factors on the therapeutic effect of HMME-PDT. Moreover, we evaluated the treatment efficacy of HMME-PDT by objectively analyzing the changes in epidermal thickness, ectatic deformed vessel diameter and depth using OCT for the first time.

## Methods

### Participants

From February 2019 to March 2021, 211 patients (114 male and 97 female, age range: 1–58 years) with Fitzpatrick skin type III–IV and clinically confirmed PWS were recruited. The treatment interval was 2–3 months. According to the facial and cervical anatomical position, lesions were divided into “central face”, “lateral face”, “neck” and “mixed parts” groups. Regarding previous PDL treatment history, lesion regression rate of <30% over 1–22 sessions of PDL treatment was defined as unresponsive to PDL and improvement degrees of more than 30% as responsive to PDL according to the criteria of Goh et al. (16). Treatment history was classified into “no treatment history”, “effective treatment” and “ineffective treatment” groups. The study protocols were approved by the Ethics Committee of The Union Affiliated Hospital of Fujian Medical University in Fujian, China. Written informed consent was obtained from all patients or their parents.

### Inclusion Criteria

The criteria for inclusion were as follows: (1) patients with PWS who met the diagnostic criteria with lesions located on the face and neck, and (2) no history of PDT, PDL or other treatments in the 3 months preceding the study period.

### Exclusion Criteria

The criteria for exclusion were as follows: (1) other concomitant skin diseases (e.g., severe acne, contact dermatitis, suppurative infection) at the site of the lesion that could affect evaluation of efficacy, (2) accompanying vascular malformations or syndromes, (3) allergy toporphyrins and analogs, (4) photosensitivity, (5) allergic constitution and scar diathesis, (6) patients with heart, liver, kidney, chronic, and other systemic diseases or those on anticoagulants, (7) pregnancy and lactation, (8) patients who participated in other drug clinical trials within 4 weeks prior to treatment, (9) patients requesting to withdraw from the study, (10) cases where no timely follow-up was performed 2–3 months after HMME-PDT treatment, (11) lost interviewees in the process of research, and (12) incomplete statistical data collection.

### Therapeutic Drugs and Methods

HMME (sterile, lyophilized powder) was supplied by Shanghai Fudan-Zhangjiang Biopharmaceutical Co., Ltd. (Shanghai, China; pharmaceutical batch number H20120076). Pre-treatment examination and preparation included tests on hepatic function, renal function, routine blood parameters and electrocardiography. Levels of all indicators were expected to be normal before treatment. Clinical images were collected using a fixed digital camera in a professional studio. The treatment site was cleaned and the surrounding skin area carefully covered. HMME was intravenously injected steadily at a dose of 5 mg/kg for 20 min. Ten minutes after the onset of injection, the lesion was subjected to 532-nm green LED light (LED Therapeutic Machine, LED-IE, Wuhan YaGe Optic and Electronic Technique Co. Ltd., Wuhan, Hubei, China, power density: 80–100 mW/cm^2^, fluence: 96–120 J/cm^2^, spot size: 10 cm, output wavelength: 532 ± 10 nm) until the end of the injection period.

### End of Treatment Observation

During the irradiation process, changes in the irradiation area were closely surveyed, irradiation time was accurately controlled, and output power monitored. The endpoint of treatment was mild or moderate swelling of skin lesions, which turned dark purple in color.

### Post-treatment and Follow-Up

After irradiation, a cold spray instrument was utilized at the treatment site for 30 min to relieve postoperative skin inflammation and an intermittent ice compress administered to relieve pain where necessary. Patients were informed of precautions, in particular, avoiding direct exposure of skin and eyes to sunlight or strong indoor light sources within 14 days after treatment and avoidance of repeated friction and scratching of the local skin area. In case mild or moderate swelling occurred, no special treatment was required. Patients were instructed to send lesion images through the WeChat and PWS consulting platforms regularly for follow-up and documentation of the relative therapeutic response.

### OCT Measurement Evaluation

**Figure 1:**
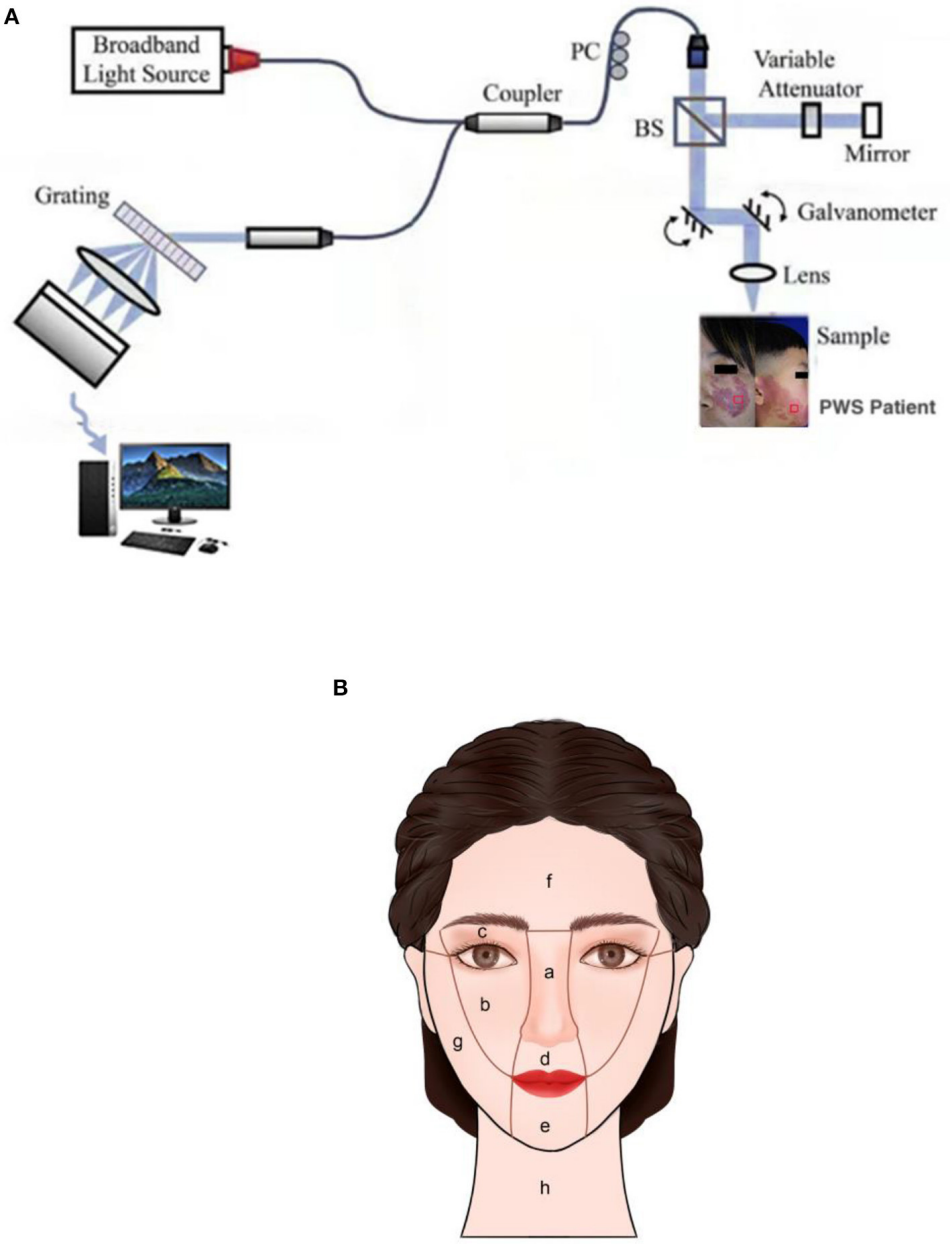
**(A)** Structure diagram of OCT system. **(B)** Anatomical division of face and neck.

A spectral domain OCT system (GAN520C1, Thorlabs Inc., Newton, USA) with a light source centered at 900 nm was employed. This system records 90 frames per second with axial and transverse resolution of 3 and 4 μm in skin tissue, respectively, scanning range of 2.0 × 1.4 mm (axial x lateral), image size of 1024 x 2256 pixels (axial x lateral), and signal-noise ratio of 102 dB. The structure of the OCT system is depicted in Figure 1A. The sample arm was packaged into a contact handle through which measurements of PWS were completed. OCT data were collected at lesions of patients from a 1 cm x 1 cm area with uniform appearance on the contralateral normal areas. The skin lesions of each patient were measured at three points and each test site evaluated twice. The average value of measurements was used to represent epidermal thickness, vessel diameter and vessel depth of PWS skin in this area. Data were collected before HMME-PDT and during the second visit at 2-3 months after treatment. In the process of data collection, a digital camera and standard anatomical position were used to record the locations of collection points to ensure consistency of measurement areas before and after treatment.

### Efficacy Criteria

Before and after each treatment, standardized digital photographs were obtained from three different angles (90° and 45° to the left and right of the treated surface) using consistent camera settings (EOS 700D; Canon, Tokyo, Japan) under the same light. Three dermatologists who were not involved in the research independently compared the changes in lesion area and color of PWS before and after two continuous HMME-PDT sessions and standard classification of quartile percentages (Table 1) was employed to evaluate efficacy (17). An outcome was considered only when all three doctors reached an agreement and in cases where no agreement was initially obtained, reevaluation was conducted until a consensus was reached.

**Table 1:** Four-level scale response evaluation.

	**Degree of improvement**	**Degree of regression of lesions (%)**
0	No improvement	0
1	Poor	1–25%
2	Moderate	26–50%
3	Good	51–75%
4	Excellent	>75%

Facial PWS was divided into central and lateral facial parts and specifically classified according to the partitioning method described by Han and co-workers (4) (Figure 1B). Mixed lesions of the central and lateral regions or lateral region and neck were defined as mixed parts. Clear images of the same patient in the same place under consistent light and angle conditions, including pre-treatment and corresponding post-treatment photographs, were selected to evaluate efficacy for each case after treatment using a quartile scale, with effective rates equal to the percentage of excellent, good, and moderate (18).

### Statistical Analysis

SPSS 22.0 statistical software was used for data analysis. Continuous variables were expressed as median (least-maximum) and categorical variables as numerical (percentage) values. The Wilcoxon signed-rank test was applied to evaluate the relationship between efficacy and treatment sessions. The Chi-square test was performed to explore associations between efficacy and gender, lesion location and treatment history and the Mann-Whitney U test conducted to evaluate the correlation between efficacy and age. Binary multivariate logistic regression analyses were performed to assess the factors independently correlated with efficacy. Paired sample t- test was applied to evaluate the changes in epidermal thickness, deformed vessel diameter and depth of PWS lesions before and after the first session of HMME-PDT, with data expressed as mean ± standard deviation. *P*-values < 0.05 were considered statistically significant.

## Results

### Efficacy of HMME-PDT in Treatment of PWS

**Figure 2:**
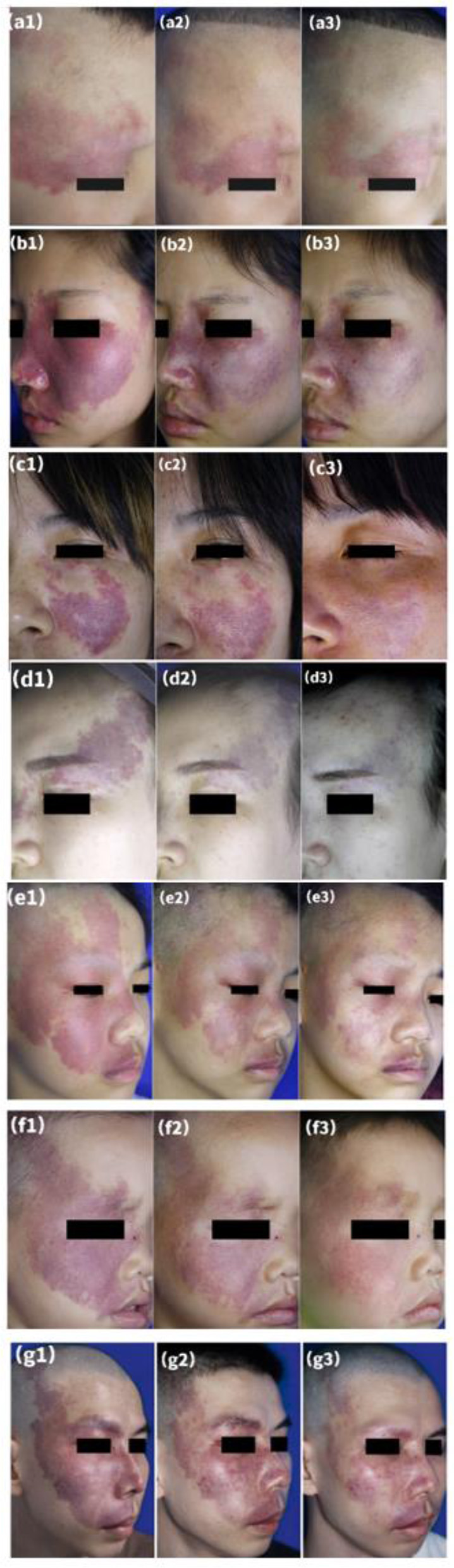
Representative examples of treatment response after two sessions of HMME-PDT. A port-wine stain in a lateral face **(a1)** before treatment, **(a2)** after one treatment, and **(a3)** after two sessions, with “good improvement” assessed after HMME-PDT **(a1–a3)**. A port-wine stain who had previously undergone two sessions of PDL without improvement **(b1)**, **(b2)** after one treatment, and **(b3)** after two sessions, with “good improvement” assessed after HMME-PDT **(b1–b3)**. A port-wine stain who had previously undergone four sessions of PDL with little improvement **(c1)**, **(c2)** after one treatment, and **(c3)** after two sessions, with “excellent improvement” assessed after HMME-PDT **(c1–c3)**. A port-wine stain who had previously undergone six sessions of PDL with little improvement **(d1)**, **(d2)** after one treatment, and **(d3)** after two sessions, with “excellent improvement” assessed after HMME-PDT **(d1–d3)**. Three port-wine stains in mixed facial regions **(e1,f1,g1)** before treatment, **(e2,f2,g2)** after one treatment, and **(e3,f3,g3)** after two treatments, with “excellent improvement” assessed at both time points **(e1–g3)**.

Clinical data obtained from patients are presented in Table 2. After the first session, 107 patients (51.7%) had a lesion regression rate >25%, after the second session, 170 patients (81.5%) had a lesion regression rate >25%. The clinical efficacy of two sessions of HMME-PDT was significantly higher than that of a single session (Z = −7.937, *P* < 0.001). Typical clinical images of partial patients before HMME-PDT and after one and two sessions of HMME-PDT are compared in Figure 2.

**Table 2:** Patients data.

**Independent variables**		**Number** ***n*** **(%)**
Age	5 (3, 14, 1–58)*	
Sex	Male	114 (54.0%)
	Female	97 (46.0%)
Lesion distribution	Middle face	66 (31.3%)
	Lateral face	45 (21.3%)
	Neck	7 (3.3%)
	Mixed parts	93 (44.1%)
History of treatment	None	96 (45.5%)
	Effective treatment group	62 (29.4%)
	Ineffective treatment group	53 (25.1%)

* *Median (P25, P75, range)*.

Under univariate and multivariate analyses, in the group administered the first session of HMME-PDT, age, lesion distribution and treatment history were independently correlated with efficacy (Tables 3, 4). Notably, efficacy was decreased with increasing age. The improvement effect on central facial lesions was greater than that on lateral lesions, while no significant differences were evident between the middle face and neck and mixed parts groups. Moreover, the efficacy of the first session of HMME-PDT on the group with no treatment history was greater than that on the effective and ineffective treatment groups.

**Table 3:** Univariate analysis of the efficacy assessment after one session of HMME-PDT.

**Independent variables**		**Effective** ***n*** **(%)**	**Non-effective** ***n*** **(%)**	**z/χ^2^**	***P***
Age				−2.612	0.009
Sex				0.273	0.601
	Male	57 (50.0%)	57 (50.0%)		
	Female	52 (53.6%)	45 (46.4%)		
Lesion distribution				13.740	0.003
	Middle face	29 (43.9%)	37 (56.1%)		
	Lateral face	34 (75.6%)	11 (24.4%)		
	Neck	3 (42.9%)	4 (57.1%)		
	Mixed parts	43 (46.2%)	50 (53.8%)		
Treatment history				22.023	<0.001
	None	66 (68.8%)	30 (31.2%)		
	Effective treatment group	20 (32.3%)	42 (67.7%)		
	Ineffective treatment group	23 (43.4%)	30 (56.6%)		

**Table 4:** Multivariate analysis of the efficacy assessment after one session of HMME-PDT.

**Independent variables**	**Waldχ^2^**	***P***
Age	−0.036	0.023
Sex	0.475	0.137
Lesion distribution		0.017
Middle face vs. Lateral face	1.346	0.003
Middle face vs. Neck	−0.187	0.832
Middle face vs. Mixed parts	0.119	0.731
Treatment history		0.001
None vs. Effective treatment group	−1.238	0.001
None vs. Ineffective treatment group	−0.970	0.012

Univariate and multivariate analyses of groups administered the second session of HMME-PDT disclosed age as an independent correlative factor for efficacy, but not gender, lesion distribution or treatment history (Tables 5, 6).

**Table 5:** Univariate analysis of the efficacy assessment after two sessions of HMME-PDT.

**Independent variables**		**Effective**	**Non-effective**	**z/χ^2^**	***P***
		***n*** **(%)**	***n*** **(%)**		
Age				−2.977	0.003
Sex				0.471	0.492
	Male	91 (79.8%)	23 (20.2%)		
	Female	81 (83.5%)	16 (16.5%)		
Lesion distribution				6.530	0.088
	Middle face	52 (78.8%)	14 (21.2%)		
	Lateral face	42 (93.3%)	3 (6.7%)		
	Neck	5 (71.4%)	2 (28.6%)		
	Mixed parts	73 (78.5%)	20 (21.5%)		
Treatment history				10.238	0.006
	None	86 (89.6%)	10 (10.4%)		
	Effective treatment group	43 (69.4%)	19 (30.6%)		
	Ineffective treatment group	43 (81.1%)	10 (18.9%)		

**Table 6:** Multivariate analysis of the efficacy assessment after two sessions of HMME-PDT.

	**Waldχ^2^**	***P***
Age	−0.035	0.034
Sex	0.489	0.214
Lesion distribution		0.288
Treatment history		0.093

### Optical Coherence Tomography Imaging of PWS Skin Lesions After HMME-PDT Treatment

**Figure 3:**
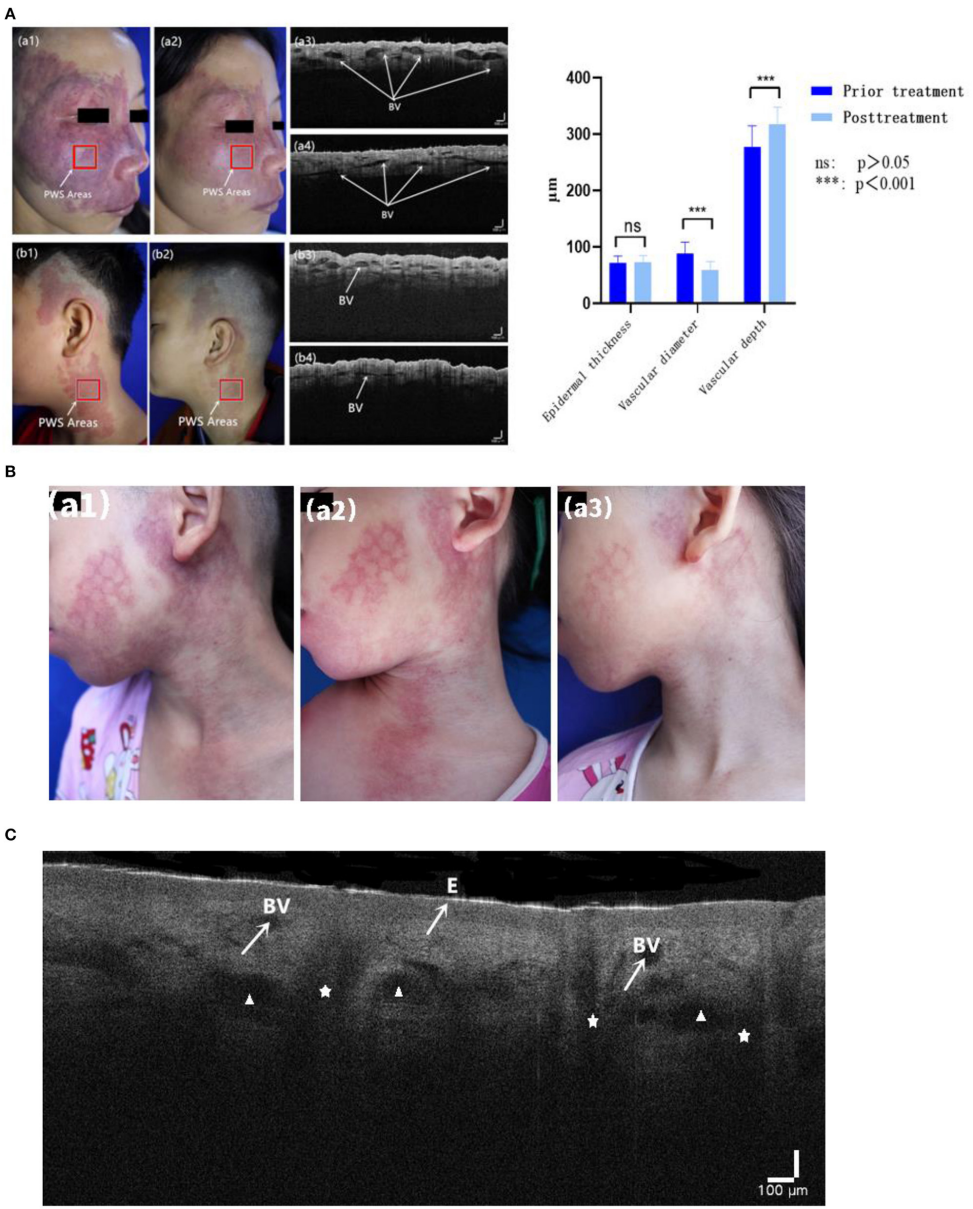
**(A)** Typical cases of HMME-PDT evaluated with OCT before and after one session of HMME-PDT (BV: dilated blood vessels). **(B)** A child left with mottled scar after PDL treatment (a1), with poor curative effect after one HMME-PDT treatment (a2), but obvious regression of skin lesions after two HMME-PDT treatments (a3). **(C)** OCT images show epidermis (E), cross section of dilated blood vessels (BV), sebaceous glands (Δ), and hair follicles (✰).

No significant differences in epidermal thickness between PWS lesions or PWS lesions and contralateral areas were evident before and after treatment (*Z* = −1.131, *P* = 0.258; *Z* = −1.445, *P* = 0.148). However, we observed marked differences in vascular diameter and depth changes between the pre- and post-treatment groups (*Z* = −5.232, *P* < 0.001; *Z* = −5.232, *P* < 0.001) (Table 7; Figure 3A).

**Table 7:** Epidermis thickness of PWS lesions and diameter and depth of ectatic vessels (*n* =36, mean±SD).

**Parameter**	**Contrast**	**Prior treatment**	**Post-treatment**	z*	P*
Thickness of epidermis (um)	73.11 ± 11.68	72.20, 11.77	72.72, 11.85	−1.131	0.258
Diameter of blood vessels (um)	rarely-seen	90.97, 25.75	59.50, 14.66	−5.232	<0.001
Depth of blood vessels (um)	rarely-seen	277.93, 36.91	317.84, 30.03	−5.232	<0.001

* *Statistics before and after HMME-PDT treatment*.

### Treatment and Adverse Reactions

All patients experienced pain and burning sensation during treatment. Follow-up visits to patients were conducted through WeChat and PWS consulting platforms with the aim of observing and recording adverse reactions to therapy. Following the first session of treatment, all patients developed localized edema, which generally lasted 1–7 days. Overall, 25.1% experienced pruritus after treatment, lasting 3–5 days. Pain occurred after treatment over 1–4 days. Overall, 4.7% patients developed small blisters in the treated area for 1–7 days, 33.6% developed scabs that fell off in 2–3 weeks, and 28.9% showed pigmentation, which subsided within 3–6 months under conditions of light avoidance.

After the second session, all patients developed localized edema, lasting 1–7 days. Overall, 23.2% had pruritus after treatment for 3–5 days, 57.8% complained of pain for 1–4 days, 3.8% displayed small blisters in the treated area lasting 1–7 days, and 29.9% developed scabs, which fell off within 2–3 weeks. Pigmentation appeared in 25.1% of patients and subsided within 3–6 months after light avoidance. No other adverse reactions, such as hypopigmentation, infection, scarring, photosensitive reaction and systemic symptoms, were documented in all cases. Postoperative adverse reactions and their incidence are presented in Table 8. Laboratory findings (including routine blood, urine, liver and renal function tests as well as electrocardiogram data) were also within normal limits.

**Table 8:** The incidence of postoperative adverse reactions.

**Adverse reactions**	**Incidence (%)**	
	**One session**	**Two sessions**
Localized edema	100%	100%
Pruritus	53 (25.1%)	49 (23.2%)
Pain	128 (60.7%)	122 (57.8%)
Small blisters	10 (4.7%)	8 (3.8%)
Scab	71 (33.6%)	63 (29.9%)
Pigmentation	61 (28.9%)	53 (25.1%)
Hypopigmentation, infection, scar	0.00%	0.00%
Photosensitive reaction, systemic symptoms	0.00%	0.00%

## Discussion

The improvement effect of two continuous HMME-PDT sessions was significantly higher than that of single treatment in our study, suggesting that the number of treatments is positively correlated with efficiency, potentially due to cumulative effects. Multiple treatments increased the overall flux and efficacy of phototherapy. Therefore, if the expected effect is not achieved after the first treatment, at least two HMME-PDT sessions are recommended before termination of therapy.

In multivariate analysis, efficacy decreased with increasing age following single treatment, which could be attributed to a number of factors. Firstly, the thinner skin of children is more conducive to light penetration. Moreover, the color of skin lesions increased with age, along with thickening and appearance of nodules and skin lesions were transformed from pink to purple in color. Our experiments revealed a close correlation of age with type of PWS lesion. Accordingly, PWS lesion type was not included in the analysis of relevant factors associated with efficacy.

Additionally, the therapeutic effect on central facial lesions was lower than that on lateral lesions, which could be due to the histological manifestations of different sites. Specifically, lesions in the center of the face are large in diameter and deep (19) with high abundance of sebaceous glands. Vessels with large diameters have less singlet oxygen on average and may therefore be harder to destroy. Savas et al. (20) proposed that the abundant parts of sebaceous glands and other appendages reduce and hinder the energy of light sources. Therefore, the issue of whether the initial treatment can sufficiently increase the light dose and energy for central facial lesions requires further investigation with larger sample sizes in the future.

In this study, no obvious differences were detected between effects on the central facial region and neck and the potential influence of small sample size on the results cannot be excluded. The lack of significant differences in therapeutic effects between the midface and mixed part groups was speculated to be due to the large lesion areas in the latter group (10). Although two light spots were used in the treatment process for patients with large lesion areas and the existing HMME-PDT 532 nm LED plane light source covered an adequate area, the level of coverage was insufficient. It may be prudent to recommend scalable and flexible LED light sources in the future, which can effectively fit the anatomical curves of the human body to achieve optimal light coverage.

In the current investigation, we explored the efficacy of HMME-PDT in patients with no history of PDL treatment, effective PDL and ineffective PDL treatment groups for the first time. Our data showed higher efficacy of therapy for patients with no treatment history relative to the other two groups. This finding may be attributable to a number of reasons. First, vessels with no treatment history were located superficially while those from groups with PDL treatment history were removed. Moreover, vessels from the ineffective treatment group displayed higher blood flow along with larger diameter and depth (21). Secondly, several patients had scars after PDL treatment and the dense hyperplasia of dermal collagen fibers could influence penetration of the light source (22). Thus, PWS lesions with deep blood vessels, dense proliferation of collagen fibers in the dermis and rich sebaceous glands respond poorly to HMME-PDT due to limited light source penetration. This phenomenon also reflects the fact that inadequate light source penetration is a significant limitation of HMME-PDT therapy. Molecular optical clearing agents may be utilized to enhance the depth of illuminant penetration (23) or we may look forward to a longer wavelength laser (755 nm or 1,064 nm) to PDT prospectively (24).

The response rate of patients from the ineffective treatment group was acceptable, which could be explained by several potential factors. First, distinct from the photothermal effect of traditional PDL, HMME-PDT targets the vasoganglion through simultaneous intracapillary photochemical and photothermal reactions (10). Furthermore, PDL damages blood vessels by heating hemoglobin. In medium blood vessels with a diameter larger than 20 μm, PDL causes coagulation *via* a photothermal effect while photocoagulation is difficult in small superficial blood vessels. Unlike PDL, HMME-PDT destroys whole blood vessels by targeting damage to the malformed blood vessel walls of PWS and contributes to the relatively thin distended blood vessels (25). Finally, VEGF is one of the known indispensable regulators of angiogenesis and HMME-PDT inhibits endothelial cell proliferation through the VEGF/Akt/mTOR pathway (26). Vascular dilation is reported to be inhibited *via* downregulation of VEGF and VEGFR mRNA (27). Interestingly, expression of VEGF is increased after PDL exposure (28), which may be the contributory factor in regeneration and revascularization of blood vessels post-treatment.

In multivariate analysis, after the second session of HMME-PDT, age was the only notable influencing factor in relation to efficacy, and efficacy decreased with increasing age. Interestingly, in terms of treatment history, favorable efficacy was achieved for all three categories of patients, suggesting no effect of PDL treatment history on efficacy of the dual HMME-PDT treatment regime. One hypothesis to explain this finding is that with repeated application, HMME-PDT improves perivascular collagen fibroplasia remaining after PDL treatment, leading to increased penetration of the 532 nm light source. Song et al. (29) previously investigated a PWS patient with a facial hypertrophic scar. In their study, PWS lesions were eliminated concurrently and the scar significantly improved after several treatments with HMME-PDT. Cai et al. (30) further demonstrated that HMME-PDT could induce apoptosis of scar fibroblasts and stimulate production of caspase-3, a key enzyme in cell apoptosis. Consistently, the group of Cui (31) showed that HMME-PDT promotes fibroblast apoptosis and reduces vascular density and scarring in nude mice. The cumulative effect of two consecutive HMME-PDT sessions, which achieved good results in patients with PDL treatment history, should also be considered. As shown in Figure 3B, a child was left with mottled scar after PDL treatment. After the first session, the improvement effect was poor while after the second session, the lesion was significantly ameliorated.

While HMME-PDT presents an effective method for treatment of PWS, non-invasive, practical and reproducible strategies for evaluation of efficacy are currently lacking.

The VISIA-CR^TM^ system is only suitable for detection of facial lesions on account of the specialist structure of the equipment and cannot effectively display thickened lesions. Dermoscopy is not available for quantitative analysis. High-frequency ultrasound (HFUS) has a high penetration depth (up to 3.7 mm) but limited resolution (32), which restricts its ability to evaluate minor changes in skin tissue. In contrast, reflectance confocal microscopy (RCM) has a lateral cell resolution of 1 μm and axial resolution of 5 μm but a shallow penetration depth (only 0.2 mm) (33). Laser speckle contrast imaging (LSCI) and laser doppler perfusion imaging (LDPI) can dynamically detect and feedback microcirculation blood flow and perfusion rate in the affected PWS area. However, these methods require a high degree of patient cooperation. Collection of images of young children is often difficult and local hemodynamic changes of some PWS lesions are not obvious. Consequently, the accuracy is poor and clinical application is limited (34).

As an alternative evaluation modality, OCT is a newly emerging non-invasive tool for visualizing skin structures at a greater depth than RCM while maintaining a resolution exceeding that of HFUS. Here, we quantitatively evaluated the morphological features of PWS blood vessels using OCT. Hemoglobin absorbs light due to the large variations in optical properties between blood and surrounding skin. Consequently, distended deformed blood vessels appear as signal-poor round or oval dark shadowed areas in the dermal papilla in OCT images (35). The deformed vessel diameter of PWS is generally >50 μm (35) and axial resolution of OCT is ~3 μm. Therefore, the outlines of ectatic deformed vessels are clearly observed. Other accessory structures in skin tissue, such as hair follicles and sebaceous glands, can be identified based on their specific morphology (Figure 3C). Several regular and relatively small linear structures exist in the dermis (generally <30 μm in width), which are observed in both normal tissue and PWS lesions and considered normal lymphatic vessels or small vessels. The diameters and depths of all malformed vessels in OCT images are averaged.

In this research, the mean vascular diameter measured prior to treatment in 36 PWS patients was consistent with data from previous RCM studies (33). Compared with histopathological findings reported in the literature, the average depth of blood vessels measured *via* OCT was more shallow. Barsky et al. (36) recorded the average depth of blood vessels in PWS *via* skin pathology biopsy of 100 patients as 460 μm. The phenomenon may be attributed to the fact that skin tissue is a high scattering medium, which limits the penetration depth of OCT. In future studies, biocompatible chemical reagents with high refractive index and high osmotic pressure, such as optical clearing agent (37), may be utilized to match the refractive index of tissue, which would reduce light scattering and enhance penetration depth. Overall, however, OCT has superior imaging depth relative to RCM.

We observed no significant differences in epidermal thickness before and after treatment, indicating that HMME-PDT does not damage the epidermal barrier at the lesion site. The overall diameter of dilated vessels was decreased significantly while vessel depth was increased, further confirming the vascular targeting ability of HMME-PDT. After the first session, the depth of deformed blood vessels increased and we attempted to increase the dose and intensity of light to achieve a better individualized effect with the next treatment. Quantitative determination of the diameter and depth of malformed blood vessels of PWS before and after HMME-PDT therapy *via* OCT provides objective data on differences in the therapeutic effect, avoids the subjectivity of human judgment by visual observation, and provides a basis for individualized and precise therapy.

OCT cannot be compared longitudinally with the clinical photograph-based quartile rating method in terms of several parameters, such as epidermal thickness, vessel diameter and depth. Furthermore, the morphological structures of malformed vessels in OCT images are mainly assessed subjectively. In the future, we plan to combine three-dimensional OCT, Doppler OCT or algorithms with OCT to verify the structures of other appendages and observe dynamic blood flow more objectively and effectively. Meanwhile, collection of more cases, design of further clinical trials, and selection of different energy density and drug doses according to diameter and depth of blood vessels within lesions should aid in determining the optimal photodynamic parameters for individual conditions of different lesions.

In conclusion, vascular targeted HMME-PDT has been shown to be effective and safe for treatment of PWS. Factors independently related to efficacy include age, lesion location and treatment history of PWS patients. Patients with younger age, perifacial areas, and no treatment history tend to show a better initial response. With increasing treatment times, age has a greater impact on effectiveness of therapy. To our knowledge, this study is the first to perform multifactorial analysis of several relevant factors both in adults and children in a large scale to explore independent factors. Meanwhile, the study firstly demonstrated the morphological structure of malformed vessels of PWS patients under OCT before and after HMME-PDT, facilitating clearer visualization and quantification for future analyses.

## Data Availability Statement

The raw data supporting the conclusions of this article will be made available by the authors, without undue reservation.

## Ethics Statement

The studies involving human participants were reviewed and approved by the Ethics Committee of The Union Affiliated Hospital of Fujian Medical University in Fujian, China. Written informed consent to participate in this study was provided by the participants or their legal guardian/next of kin. Written informed consent was obtained from the individual(s), and minor(s)' legal guardian/next of kin, for the publication of any potentially identifiable images or data included in this article.

## Author Contributions

XX and LL conceived and designed the study. YL performed the experiments. YL, WG, JK, YF, and JL collected the clinical data. YL and LL analyzed the data and wrote the paper. XX reviewed and edited the manuscript. All authors read and approved the manuscript.

## Funding

This work was funded by grants from the Fujian Province Natural Science Foundation (2020J011034).

## Conflict of Interest

The authors declare that the research was conducted in the absence of any commercial or financial relationships that could be construed as a potential conflict of interest.

## Publisher's Note

All claims expressed in this article are solely those of the authors and do not necessarily represent those of their affiliated organizations, or those of the publisher, the editors and the reviewers. Any product that may be evaluated in this article, or claim that may be made by its manufacturer, is not guaranteed or endorsed by the publisher.
